# Fission Yeast SCYL1/2 Homologue Ppk32: A Novel Regulator of TOR Signalling That Governs Survival during Brefeldin A Induced Stress to Protein Trafficking

**DOI:** 10.1371/journal.pgen.1006041

**Published:** 2016-05-18

**Authors:** Katarzyna M. Kowalczyk, Janni Petersen

**Affiliations:** 1 Faculty of Life Sciences, University of Manchester, Manchester, United Kingdom; 2 Flinders Centre for Innovation in Cancer, School of Medicine, Flinders University, Adelaide, Australia; 3 South Australia Health and Medical Research Institute, Adelaide, Australia; The University of North Carolina at Chapel Hill, UNITED STATES

## Abstract

Target of Rapamycin (TOR) signalling allows eukaryotic cells to adjust cell growth in response to changes in their nutritional and environmental context. The two distinct TOR complexes (TORC1/2) localise to the cell’s internal membrane compartments; the endoplasmic reticulum (ER), Golgi apparatus and lysosomes/vacuoles. Here, we show that Ppk32, a SCYL family pseudo-kinase, is a novel regulator of TOR signalling. The absence of *ppk32* expression confers resistance to TOR inhibition. Ppk32 inhibition of TORC1 is critical for cell survival following Brefeldin A (BFA) induced stress. Treatment of wild type cells with either the TORC1 specific inhibitor rapamycin or the general TOR inhibitor Torin1 confirmed that a reduction in TORC1 activity promoted recovery from BFA induced stress. Phosphorylation of Ppk32 on two residues that are conserved within the SCYL pseudo-kinase family are required for this TOR inhibition. Phosphorylation on these sites controls Ppk32 protein levels and sensitivity to BFA. BFA induced ER stress does not account for the response to BFA that we report here, however BFA is also known to induce Golgi stress and impair traffic to lysosomes. In summary, Ppk32 reduce TOR signalling in response to BFA induced stress to support cell survival.

## Author Summary

The Target of Rapamycin (TOR) pathway plays a central role coordinating cell growth and cell division in response to the different cellular environments. This is achieved by TOR controlling various metabolic processes, cell growth and cell division, and in part by the localisation of TOR protein complexes to specific internal endomembranes and compartments. Here, we report a novel role for the SCYL family pseudo-kinase, Ppk32 in restraining TOR signalling when cells experience stresses, which specifically affect endomembranes and compartments where TOR complexes are localised. Cells exposed to endomembrane stress (induced by Brefeldin A), displayed increased cell survival when simultaneously treated with the TOR complex 1 (TORC1) inhibitor, rapamycin, presumably because the reduction in TORC1 signalling slows cellular processes to allow cells sufficient time to recover and adapt to this stress. Importantly cancer, neuro-degeneration and neurological diseases are all associated with stress to the endomembrane protein trafficking system. Our findings suggest that therapeutic rapamycin treatment to reduce TOR signalling and block proliferation of cancer cells, which are inherently experiencing such stress, may have the unintended consequence of enhancing cell survival. It is notable, therefore, that our reported mechanisms to reduce Ppk32 protein levels, likely to be conserved in humans, may represent a way to increase TOR signalling and thus increase cell death of cancer types with inherent stress to these internal membrane systems.

## Introduction

TOR signalling allows eukaryotic cells to adapt their metabolism, cell growth, stress and survival to meet the demands of the prevailing conditions [1]. TOR kinases form at least two distinct complexes: TOR complex 1 (TORC1) and TORC2 [2–4]. These complexes are defined by the presence of unique binding partners; Raptor interacts with TOR kinase in complex 1, whereas Rictor replaces Raptor in complex 2 [2,4]. The yeasts differ from higher eukaryotes in having two separately encoded TOR kinases. In fission yeast Tor1 is the main kinase that binds Ste20 (rictor) in TORC2, whereas Tor2 is in a complex with Mip1 (raptor) in TORC1 [5–7]. Yeast and mammalian TORC1 responds to changes in the abundance of nutrients and growth factors (mammals) to adjust the cell cycle, cell growth and metabolism accordingly. The roles for TORC2 include modulation of the actin cytoskeleton [3,8], stress responses [9] and chaperone-mediated autophagy [10]. Both TOR complexes localise to membrane-enclosed structures. In nutrient rich conditions, TORC1 is found on lysosomes [11] and the Golgi apparatus [12], whereas TORC2 localises mainly to the endoplasmic reticulum (ER) [13]. TORC2 can also be found on the plasma membrane [13,14] and lysosomes [10].

The endoplasmic reticulum originates from the nuclear envelope to extend throughout the cell. ER membranes are in constant contact with the Golgi apparatus. COP-mediated trafficking, regulates vesicle transport both to and from the ER and Golgi and between Golgi stacks [15]. Once proteins reach the trans-Golgi apparatus, they are directed either to plasma membrane, lysosomes or other vesicle-based compartments. Therefore, these endomembranes function as protein and lipid factories but also as scaffolds for complexes such as TOR signalling modules.

The lactone antibiotic Brefeldin A (BFA) inhibits the GEFs for class II ARFs (ADP-Ribosylation Factor a GTPase) [16,17] to release ARF into the cytosol. This release reversibly blocks traffic between the Golgi and ER and within the Golgi stacks to generate Golgi stress. The BFA induced block to protein traffic can also induce ER stress through the unfolded protein response (UPR) [18]. Furthermore, BFA impairs traffic to the lysosomes [19]. Importantly, BFA has been key to elucidating the mechanisms of trafficking at the Golgi.

Here we report that Ppk32, a SCYL family pseudo-kinase, is a novel regulator of TOR signalling. Reduced TORC1 activity promoted survival upon Brefeldin A (BFA) induced stress. Ppk32 was critical for inhibition of TORC1 signalling and survival during this BFA induced stress. Ppk32 also controlled TORC1 activity during sexual differentiation and ablation of *ppk32*^*+*^ expression advanced differentiation. The response to BFA that we report here is not regulated though UPR induced ER stress. Therefore, cells are likely to sense Golgi stress, or impaired traffic to lysosomes, following BFA treatment. Finally we show that Ppk32 phosphorylation on two sites is important for function because it controls Ppk32 protein levels and determines cellular sensitivity to BFA induced stress and TOR inhibition. This target sites for phosphorylation are conserved through to human members of the SCYL pseudo-kinase family

## Results

### The fission yeast SCYL homologue Ppk32 regulates TOR signalling

**Fig 1:**
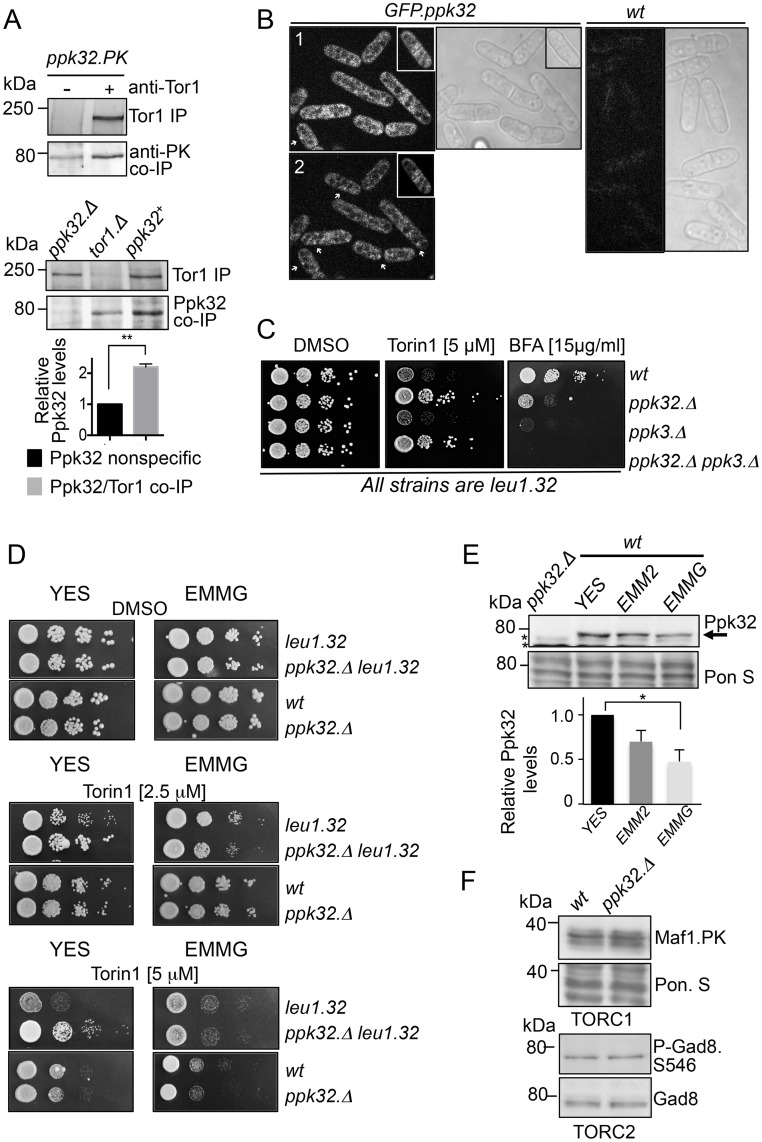
The fission yeast SCYL homologue Ppk32 regulates TOR signalling. (A) Tor1 immuno-precipitations. Soluble proteins were extracted from exponentially grown strains: *ppk32*.*PK* strain (upper) and wild-type, *ppk32*.*Δ* and *tor1 Δ* (lower). Tor1 immuno-precipites and Ppk32 co-immuno-precipites were assessed from the same gels. The relative levels of specific Ppk32 immuno-precipitation are shown. (B) Live cell imaging: GFP and transmitted light images of GFP-Ppk32 and wild type control strains. (1) shows a projection of 4 single planes covering 0.5 nm (2) shows a single plane image, → indicates cytoplasmic foci. For *wild type* cells full Z-stack projection is shown (C, D) Growth assay. Exponentially YES-grown cells of indicated strains were spotted in 10-fold serial dilution onto rich medium plates (YES) or YES supplemented with Torin1, BFA or vehicle (DMSO) alone. (E,F) Early exponential cells grown in indicated media. Samples from un-perturbed cultures were subjected to Western blot analysis to monitor Ppk32 levels (E), * indicates a background band, or Maf1.PK (upper part) or Ser546 Gad8 phosphorylation (Lower part) (F). Ponceau S staining was used as a loading control.

**Fig 2:**
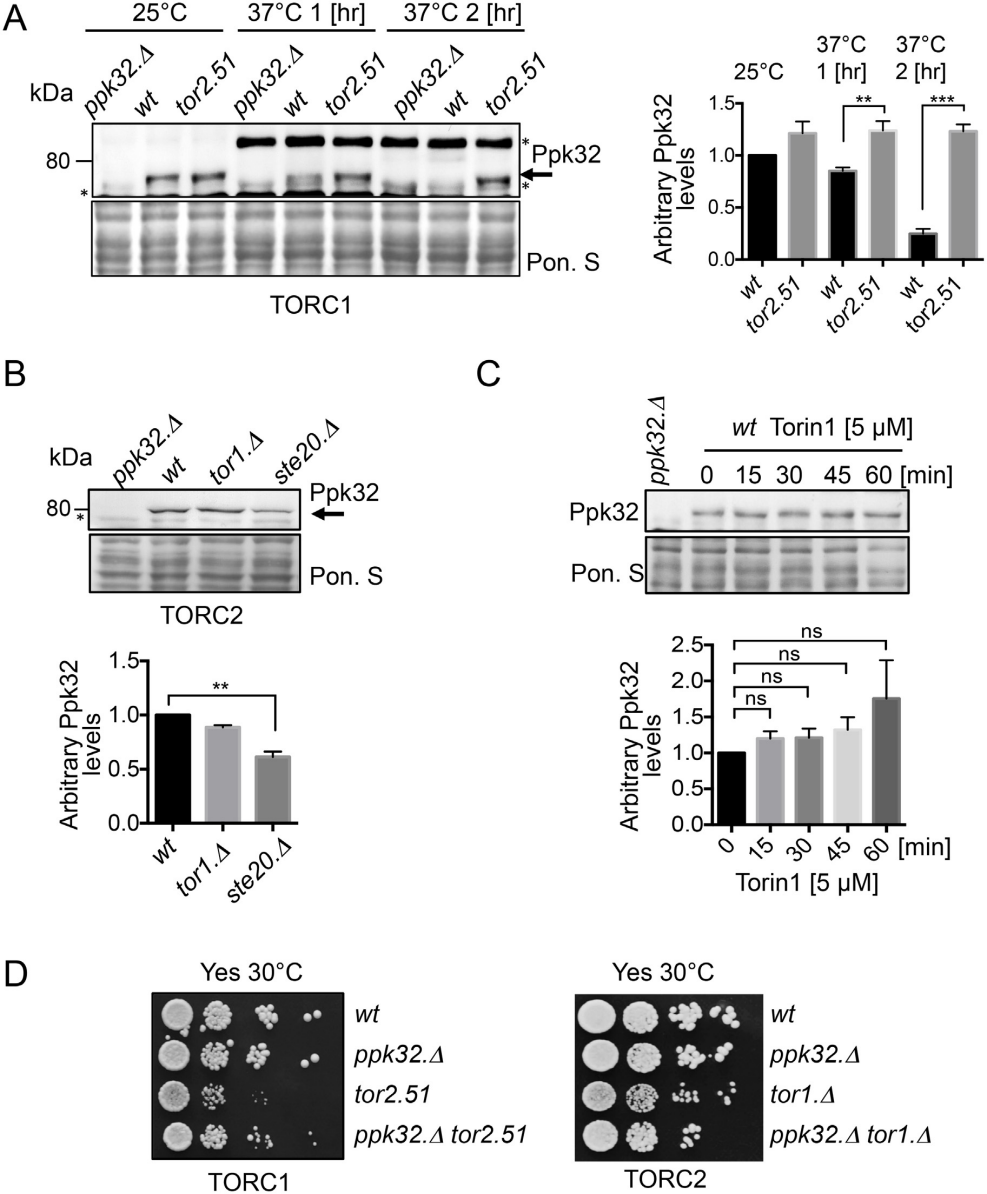
Ppk32 protein level is regulated by TOR signalling. All strains are leucine autotrophs. (A, B, C) Western blot analyses of Ppk32, * indicates a background band. Early exponential cells were grown in rich medium (YES). Samples from indicated cultures were subjected to Western blot. (A) Cells were shifted from 25°C to 37°C for indicated time. Note an unknown background band is unregulated by the heat stress. (C) Torin1 was added. Ponceau S staining was used as a loading control. (D) Growth assay. Exponentially grown cells of the indicated strains were spotted in 10-fold serial dilution onto rich medium plates (YES).

The ability of rapamycin to specifically inhibit TORC1 has facilitated extensive characterisation of TORC1; in contrast TORC2 regulation is less well defined. Preliminary data suggested that the *S*. *pombe* Ppk32 kinase co-immuno-precipitated with Tor1 (the main catalytic component of TORC2) in a large-scale screen to identify TORC2 interacting proteins. Small-scale Tor1 immuno-precipitations validated this interaction with Tor1, through co-precipitation of both Ppk32 and of PK tagged Ppk32 with Tor1 (Fig 1A). Ppk32 is a member of the SCYL pseudo-kinase family that includes the human SCYL1 and SCYL2 kinases. SCYL kinases have an amino-terminal pseudo-kinase domain and carboxy-terminal serine-rich sequences separated by HEAT repeats that are known to facilitate protein-protein interactions. TOR kinases interact with Raptor (TORC1) and Rictor (TORC2). Interestingly, this interaction is mediated, in part, through the HEAT repeats found in all 3 proteins [2]. Fission yeast TORC1 is localized to the surface of vacuoles (yeast lysosomes) and to undefined cytoplasmic foci that are not vacuoles [20]. In contrast, TORC2 localises to cell ends and to the cell equator (sites of the ER and plasma membrane) during late stages of cell division [21]. A GFP-Ppk32 strain (S1B Fig) revealed that Ppk32 was present in the cytoplasm (although signals were faint), and it was excluded from the inside of vacuoles (Fig 1B). In some places cytoplasmic Ppk32 accumulated in small foci (Fig 1B-2), between two to ten foci were seen in each cell. Ppk32 was also concentrated at the cell equator during cell division in more than 60% of cells (Fig 1B). Thus, Ppk32 localizes to structures where TORC1 and/or TORC2 may reside.

In human cells the SCYL1 kinase has been implicated in retrograde traffic between the Golgi and ER [22], a process that is affected by the lactone antibiotic Brefeldin A (BFA). BFA effectively blocks Golgi transport to compromise viability in fission yeast [23]. The fission yeast genome encodes an additional SCYL homologue, Ppk3. Strains from which the genes encoding both homologues had been deleted were more sensitive to BFA than wild type controls (Fig 1C) (and GFP-Ppk32 cells S1C Fig). Importantly, Ppk32 but not Ppk3 deficient mutants were resistant to a potent inhibitor of both TOR complexes, Torin1 [24] (Fig 1C). This Ppk32 dependent resistance to Torin1 was determined by the nutritional context, as *ppk32*.*Δ* (*ppk32*^+^ deletion) cells were only resistant to Torin1 when they were also auxotrophic for leucine and grown on nutrient rich (YES) media (Fig 1D). Neither prototrophic *ppk32*.*Δ* grown on rich YES nor auxotrophic *ppk32*.*Δ* mutants grown on minimal media were drug resistant. Therefore, unless otherwise stated, all experiments in this report use leucine autotrophic strains grown on rich YES medium.

Assessment of Ppk32 levels in total protein extracts revealed that Ppk32 protein levels in rich media were twice those of cells grown in minimal EMMG media (Fig 1E). A similar increase of Ppk32.PK total protein levels was seen in YES grown cells (S1E Fig). Addition of the Phos-tag reagent [25] to the resolving gel revealed that Ppk32.PK is phosphorylated, however similar relative levels of phosphorylation were observed in both media (S1E Fig). The increased Ppk32 total protein level in YES may explain the increased Torin1 resistance of *ppk32*.*Δ* cells compared to *ppk32*^*+*^ cells when grown in rich YES medium. Elevated TOR signalling or deficient drug uptake by *ppk32*.*Δ* mutant could account for the resistance to Torin1. The decline in TORC1 controlled Maf1 phosphorylation [26] after Torin1 treatment followed similar dosage effects, in *wt* and *ppk32*.*Δ* cells (S1D Fig) to indicate that *ppk32*.*Δ* mutants are proficient for drug uptake. Fission yeast TORC2 phosphorylates the AGC kinase Gad8 (AKT homolog) at Ser-456 [27]. The phosphorylation profiles of two TOR substrates, Maf1 and Gad8.S546 phosphorylation (Fig 1F), suggests that, while *ppk32*.*Δ* mutants cells are resistant to Torin1 (Fig 1C), Ppk32 does not appear to have a major impact on either TORC1 or TORC2 signalling under non-stressed condition.

### TOR signalling controls Ppk32 levels

To gain further insight into TOR and Ppk32 regulation, Ppk32 protein levels were assessed in TOR deficient mutants. Tor2 is the main catalytic kinase component of TORC1 [5–7]. TORC1 is essential for growth of all organisms, including fission yeast, [28,29]. We therefore incubated temperature sensitive *tor2*.*51* [5] mutants at 37°C to inactivate TORC1 activity. Ppk32 levels were maintained at 37°C (the restrictive temperature) when TORC1 was inactivated (Fig 2A). In contrast, Ppk32 levels in wild type cells were significantly reduced 2 hours following a shift to 37°C in wild type cells (Fig 2A) (note: the level of an unknown protein, recognised by anti-ppk32 antibodies in *ppk32*.*Δ* mutants, is strongly induced by heat stress). In a similar heat stress experiment of wild type cells, TORC1 dependent Maf1 phosphorylation increased 1 hour upon after shift to 37°C (S2A Fig), suggesting that TORC1 may increase 1 hr after heat stress (S2A Fig). These results suggest that Ppk32 protein turnover requires TORC1 activity. Steady state Ppk32 proteins levels were reduced in strains that were TORC2 deficient because they lacked Ste20 (the fission yeast version of the conserved TORC2 specific component Rictor) (Fig 2B), a similar decrease in Ppk32.PK total protein levels was seen in the *ste20*.*Δ* mutant (S2C Fig). No significant reduction in Ppk32 levels was observed in *tor1*.*Δ* mutants, that lack the main TORC2 kinase, however Tor2 kinase can also interact with Ste20 and this interaction is enhanced in the absence of Tor1 [30]. Simultaneous inhibition of both TORC1 and TORC2 through treatment with Torin1 did not significantly change Ppk32 levels (Fig 2C). In summary, TORC1 negatively regulates Ppk32 protein levels whilst TORC2 is required to maintain steady state levels.

### Enhanced fitness of TORC1 and TORC2 mutants following deletion of *ppk32*^*+*^

The resistance to Torin1 induced TOR inhibition in Ppk32 deficient mutant cells suggests that Ppk32 opposes TOR signalling (Fig 1C). Furthermore, TORC1 inhibition in *tor2*.*51* mutants, enhanced Ppk32 levels when compared to wild type cells (Fig 2A). We therefore used colony size as a good proxy for cell fitness [31] to ask whether Ppk32 contributes to the reduced growth rate fitness of *tor2*.*51* cells. Ppk32 removal enhanced *tor2*.*51* fitness (Figs 2D and 4A). Loss of *ppk32* also had a less pronounced, positive, impact on the fitness of *tor1*.*Δ* cells (Figs 2D and 4C). Thus, Ppk32 reduces the fitness of cells compromised for either TORC1 or TORC2 activity. Ppk32 may therefore constitute a novel inhibitor of TOR signalling. To test this possibility *ppk32*^+^ was overexpressed in wild type cells grown in minimal media to facilitate high levels of expression. Ppk32 over-expression dramatically reduced cell growth (S3A Fig) and this block to cell proliferation correlated with a decrease in the TORC1 and TORC2 dependent Maf1.PK and Gad8 S546 phosphorylation respectively (S3B Fig). In summary, elevation of Ppk32 levels reduced both TORC1 and TORC2 signalling, while abolition of *ppk32* expression provided Torin1 resistance and stimulated growth of TORC1 and TORC2 mutants.

### Decreased TORC1 signalling promotes survival during BFA induced stress

Brefeldin A (BFA) inhibits Guanine nucleotide Exchange Factors (GEFs) for ARF GTPases [16,17]. ARFs regulate COPI recruitment to Golgi membranes to facilitate retrograde trafficking within the Golgi and from the Golgi to the ER [32,33]. Importantly BFA can also induce ER stress through the unfolded protein response (UPR) and impair traffic to lysosomes [19]. We next determined whether the BFA sensitivity of fission yeast observed here (Fig 1C) arose from ER stress. In fission yeast UPR is regulated by Ire1 also known as Ppk4 [34]. The *ire1*.*Δ* mutant was not sensitive to BFA whereas double *ppk32*.*Δ ire1*.*Δ* deletion mutant was (S4A Fig). This result shows that the BFA sensitivity we observe arises from either Golgi stress or impaired traffic to lysosomes (yeast vacuoles). Interestingly, the human Ppk32 homolog the SCYL1 kinase has been implicated in retrograde traffic between the Golgi and ER [22]. SCYL1 is thought to act as a scaffold protein that recruits ARFs to COPI complexes, furthermore, BFA treatment releases ARFs from Golgi membranes in to the cytosol [35,36]

**Fig 3:**
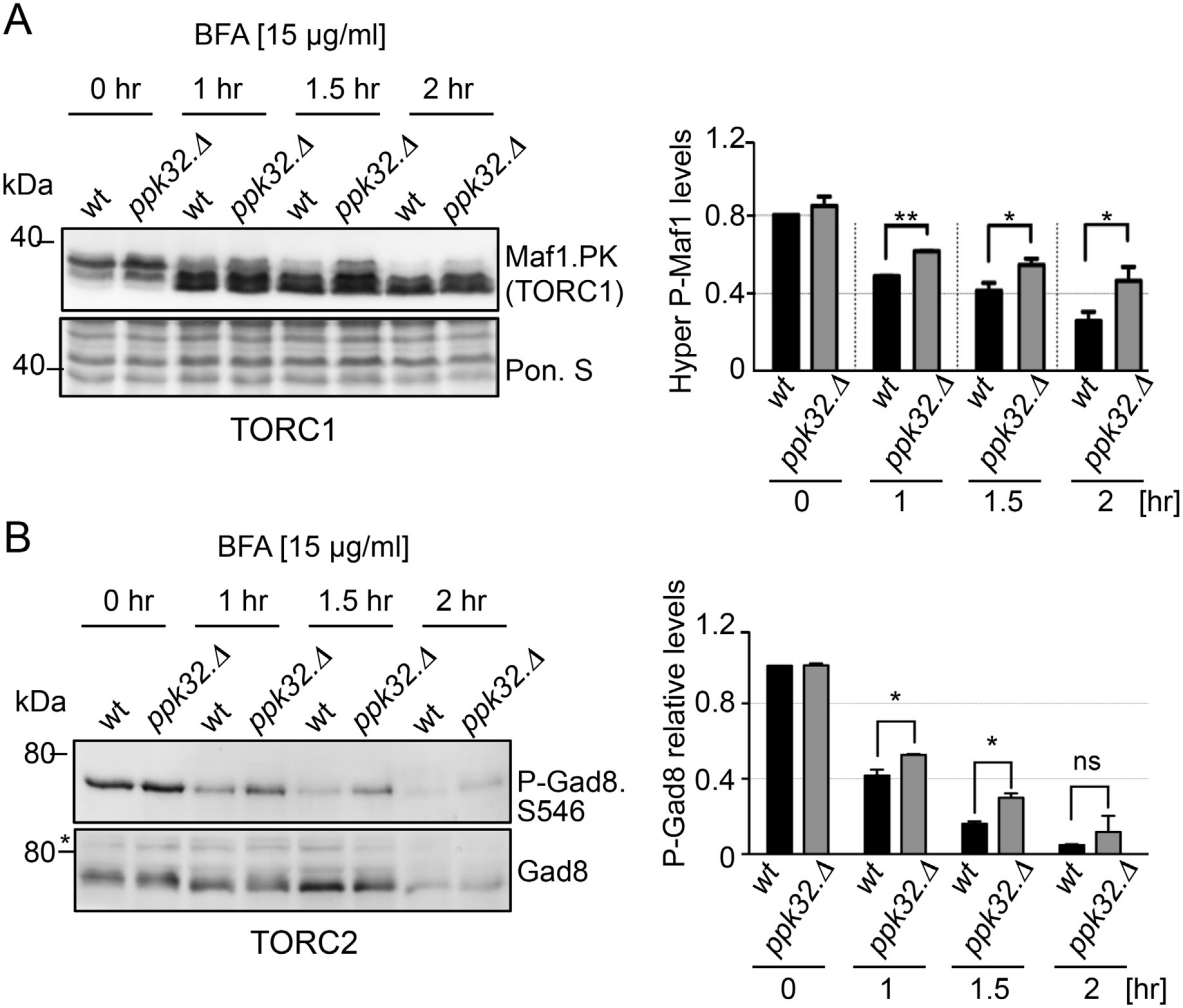
TOR signalling is reduced by BFA-induced stress. All strains are leucine autotrophs. (A, B) Early exponential cells were grown in rich medium (YES) with addition of Brefeldin A (BFA). Western blot analysis of Maf1.PK (A) or Ser546 Gad8 phosphorylation (B). Ponceau S staining was used as a loading control.

**Fig 4:**
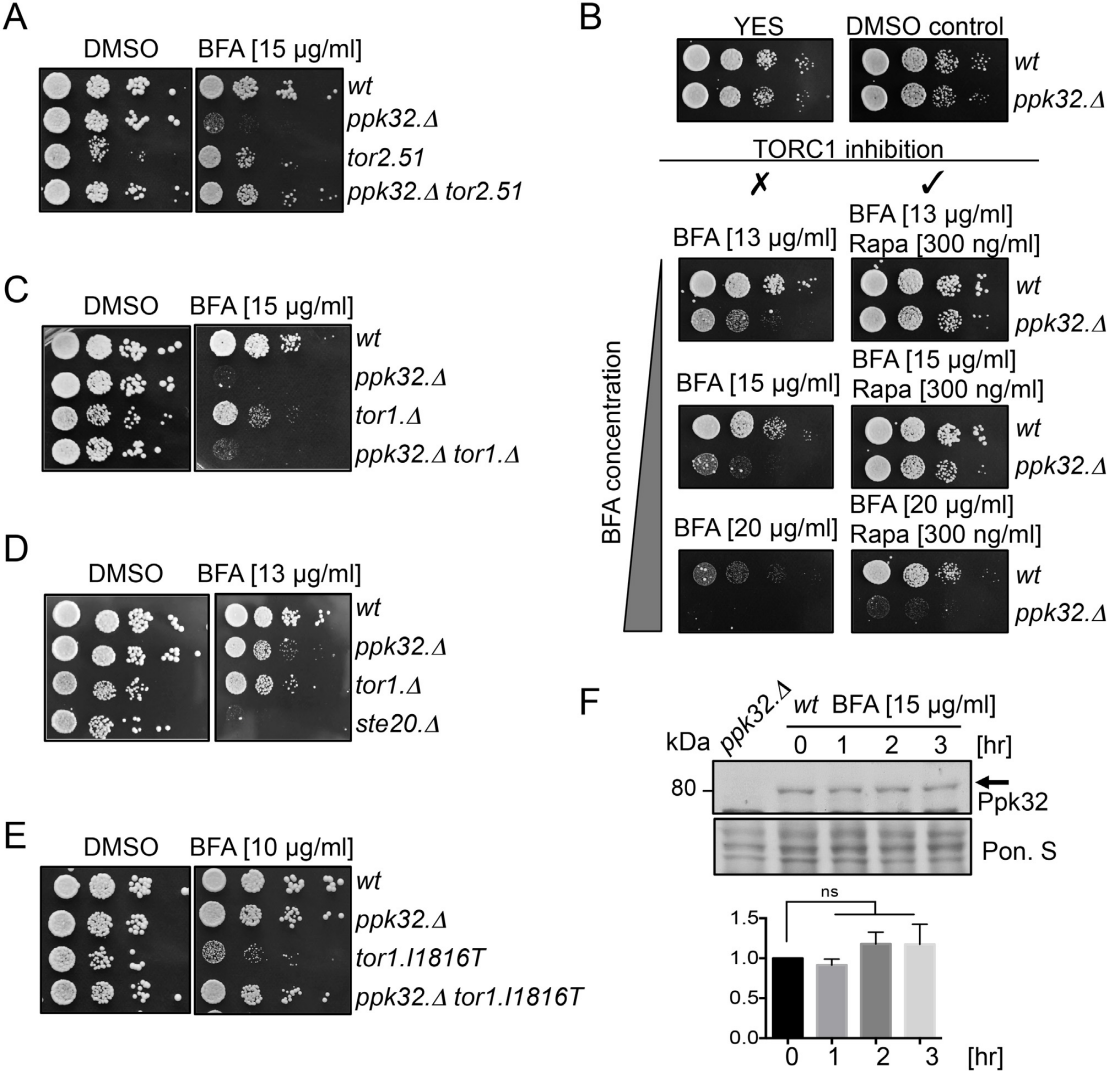
Decreased TORC1 signalling promotes survival during BFA-induced Golgi stress. All strains are leucine autotrophs. (A-E) Growth assays. Exponentially grown cells of the indicated strains were spotted in 10-fold serial dilution onto rich medium plates (YES) supplemented with BFA, BFA and rapamycin or vehicle (DMSO) alone. (F) Western blot analysis of Ppk32 levels. Early exponential cells were grown in rich medium (YES) with addition of Brefeldin A (BFA). Ponceau S staining was used as a loading control.

We next assessed the impact of BFA on TOR signalling in wild type and Ppk32 deficient cells. Previous work established that BFA treatment reduced mTORC1 activity [37]. In wild type fission yeast cells both TORC1 and TORC2 activity were reduced by BFA treatment (Fig 3A and 3B). Importantly, in *ppk32*.*Δ* mutants, the BFA induced TOR inhibition of both complexes was less pronounced to suggest that the BFA sensitivity of *ppk32*.*Δ* mutants (Fig 1C) might arise from increased TOR activity in this mutant. To test this hypothesis, we generated a *ppk32*.*Δ tor2*.*51* double mutant in which TORC1 signalling would be reduced by the mutation in Tor2. The *ppk32*.*Δ tor2*.*51* double mutant was less sensitive to BFA than the single *ppk32*.*Δ* mutant alone (Fig 4A) to imply that reduced TORC1 signalling may promote survival upon exposure to BFA induced stress [26]. To further test this hypothesis rapamycin was added to BFA treated cells. Importantly although rapamycin treatment does reduce TORC1 activity (S2B Fig), the reduction in activity is not sufficient to block cell growth in fission yeast [38]. Addition of rapamycin to inhibit TORC1 activity in *ppk32*.*Δ* mutants abolished their sensitivity to 15μg ml^−1^ BFA (Fig 4B). Furthermore, inhibition of TORC1 in *wild type* cells with rapamycin promoted growth at a higher BFA concentration (20μg ml^−1^) (Fig 4B).

Rapamycin inhibits TORC1 activity when in a complex with FKBP12 [3] (known as Fkh1 in fission yeast [29]). To determine whether the impact of Rapamycin on BFA sensitivity was an indirect one of drug interference in which rapamycin affected BFA uptake, and thus bioavailability, we exposed *fkh1*.*Δ* mutants to BFA stress. However, the fact that, in contrast to wild type cells, rapamycin did not enhance growth of BFA treated *fkh1*.*Δ* mutants (S5A Fig), excludes the possibility that rapamycin blocked BFA uptake. We therefore conclude that it is the reduction in TORC1 activity that promotes growth upon BFA induced stress.

Addition of Torin1 to inhibit both TORC1 and TORC2, also enhanced cell survival during BFA induced stress (S5B Fig). 20μg ml^−1^ BFA blocked growth of *ppk32*.*Δ* mutants. Addition of Torin1, but not rapamycin, liberated this block to cell growth (Fig 4B, S5B Fig). As mentioned previously, rapamycin reduces but does not completely block TORC1 activity (S2B Fig), whereas a complete block of TORC1 activity is achieved by treatment with Torin1 [24,26]. This may explain the increased potency of Torin1 over rapamycin in combination with BFA. Alternatively a combined reduction of TORC1 and TORC2 activity may be beneficial for survival following BFA induced stress.

We next exposed TORC2 deficient mutants to BFA to assess the role of TORC2 in BFA induced stress. Both *tor1*.*Δ* and *tor1*.*Δ ppk32*.*Δ* double mutants were sensitive to BFA and the TORC2 specific mutant *ste20*.*Δ* was hyper-sensitive to BFA (Fig 4C and 4D). Furthermore, the constitutively active TORC2 mutant *tor1*.*I1816T* [39] was also hyper sensitive to BFA (Fig 4E). This suggests that TORC2 activity is essential for cell fitness during BFA induced stress, but only at moderate levels. Interestingly, BFA resistance was restored in the *tor1*.*I1816T ppk32*.*Δ* double mutant (Fig 4E), suggesting that the hypersensitivity of the constitutively active TORC2 mutant *tor1*.*I1816T* is, at least partially, Ppk32 dependent. When considered together these observations indicate that the BFA-induced decrease, but not full inhibition, in TORC2 activity (Fig 3B) that we observed in wild type cells may be important for cell survival. Finally, neither BFA treatment nor the presence of the *tor1*.*I1816T* mutation had any impact on Ppk32 protein levels (Fig 4F, S6A Fig).

In summary, our data demonstrate that enhanced TORC1 activity accounts for the BFA sensitivity of *ppk32*.*Δ* mutants. Decreased TORC1 activity promoted cell survival and recovery from BFA induced stress.

### Ppk32 controls TORC1 signalling during sexual cell differentiation

**Fig 5:**
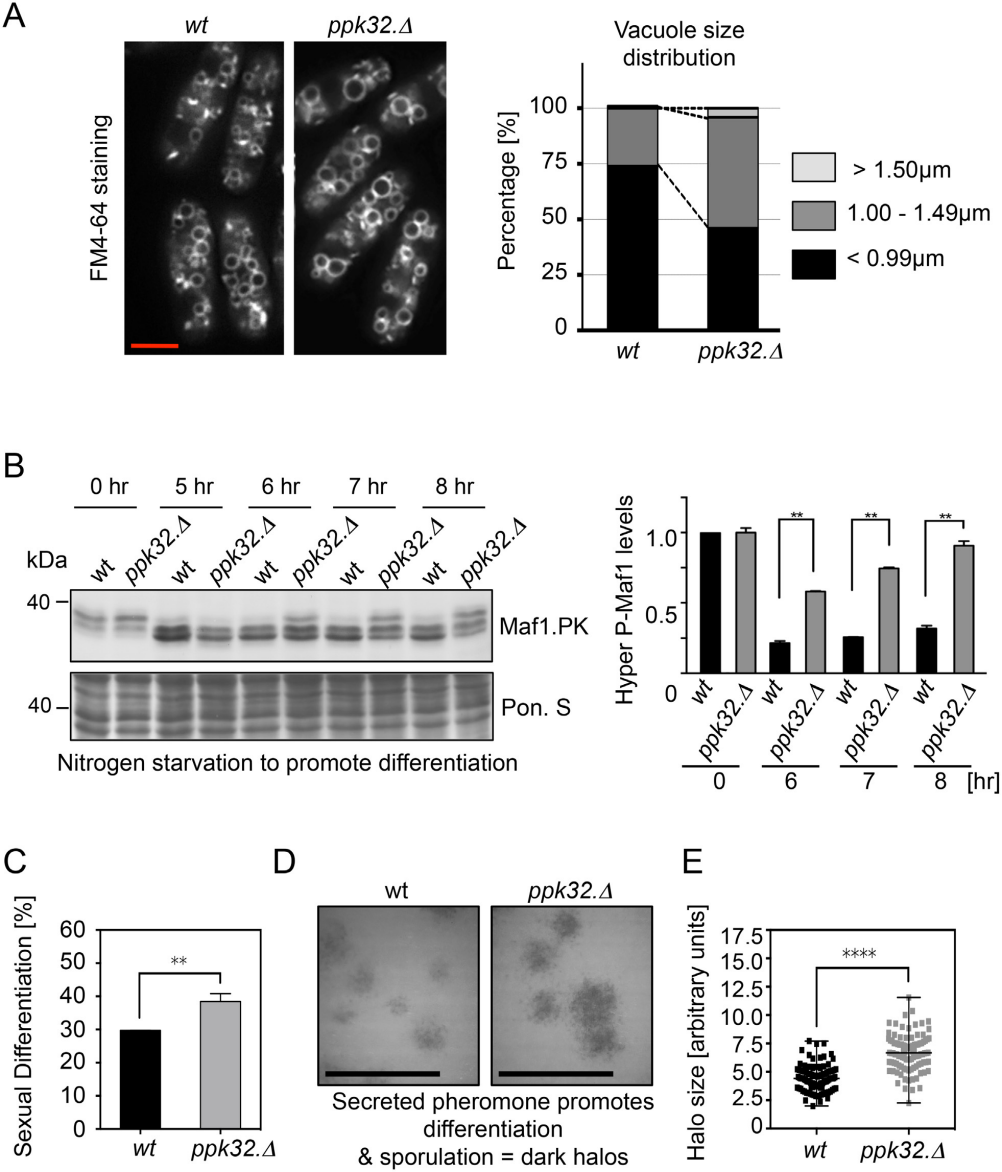
Ppk32 controls TORC1 signalling during sexual cell differentiation. All strains are leucine autotrophs. (A) Vacuole visualisation. Left-hand side: exponential cells of indicated strains that had been grown in YES were incubated with FM4-64 lipid dye for 30 min. Right-hand side: quantification of vacuole size distribution within the cultures. For each strain at least 500 vacuoles were measured. (B) Western blot analysis of Maf1.PK phosphorylation. Early exponential cells were grown in rich medium (YES) and shifted into sporulation liquid (SPL). Ponceau S staining was used as a loading control. (C) Mating efficiency. Exponentially YES-grown homothallic strains of opposite mating types were mixed in ratio 1:1 and spotted onto SPA plates. After 8 hours fused cells were counted. (D) Halo assay. Exponentially YES-grown diploid tester strain was mixed with indicated *h*^*−*^ strains in ratio 200:1 and spotted onto MSA plates. After overnight incubation sporulated cells were stained with iodine vapor. (E) Quantification of halo size distribution in cultures in D.

Human SCYL1 regulates retrograde traffic to modulate Golgi homeostasis [15,22,35], whereas SCYL2 regulates clathrin-mediated endocytosis [40,41]. Despite the high level of similarity between SCYL2 and Ppk32, *ppk32*.*Δ* cells were proficient for endocytosis. FM4-64 is a lipophilic dye that incorporates into the plasma and vacuolar membranes via endocytosis [42]. FM4-64 staining of vacuoles in *ppk32*.*Δ* mutants (Fig 5A) identified a modest increase in vacuolar size to suggest that an absence of *ppk32* expression favours vacuole fusion and/or diminishes vacuolar fission.

In the presence of cells of both mating types nitrogen starvation induces sexual differentiation to promote cell fusion, meiosis and sporulation [43,44]. Vacuoles play an important role during sexual differentiation through their participation in autophagy [45]. Autophagy deficient mutants are sterile but can complete differentiation when nutrients are resupplied [45]. High TORC1 signalling represses sexual differentiation [5,27,46]. The inhibition of TORC1 signalling, arising from nutrient starvation induces differentiation and so promotes cell fusion. Importantly however, TORC1 signalling must be reinstated to support further differentiation because diploid mutants with deficiencies in the fission yeast raptor, Mip1, are unable to execute meiosis and sporulation [47]. Thus, after its initial repression has been triggered by nutrient reduction, TORC1 is reactivated by autophagy-supplied nutrients.

The survival of cells deleted for *ppk32* following prolonged periods of nutrient deprivation indicates that *ppk32*.*Δ* mutant cells are autophagy proficient (S6B Fig). Nitrogen starvation promoted TORC1 inhibition in both *wt* and in *ppk32*.*Δ* cells (Fig 5B), however TORC1 activity returned more rapidly to *ppk32*.*Δ* cells (Fig 5B) and sexual differentiation was increased by a further 10% eight hours after starvation (Fig 5C). Pheromone secretion was also enhanced in *ppk32*.*Δ* mutant cells (Fig 5D). A “halo assay” can be used as a proxy for pheromone secretion. It deploys a diploid tester strain that cannot sporulate unless external pheromone (M-factor) is supplied [48]. Sporulating diploid tester strains can be visualized because the starch in their spore walls generates a dark stain when exposed to iodine vapour. Mixing a tester strain with *wt* or *ppk32*.*Δ* mating partners at a ratio of 200:1 revealed increased pheromone secretion from *ppk32*.*Δ* cells (Fig 5D and 5E). In summary, the presence of Ppk32 during sexual differentiation retards TORC1 reactivation to delay sexual differentiation.

### Ppk32 phosphorylation controls protein levels and function during BFA induced stress

**Fig 6:**
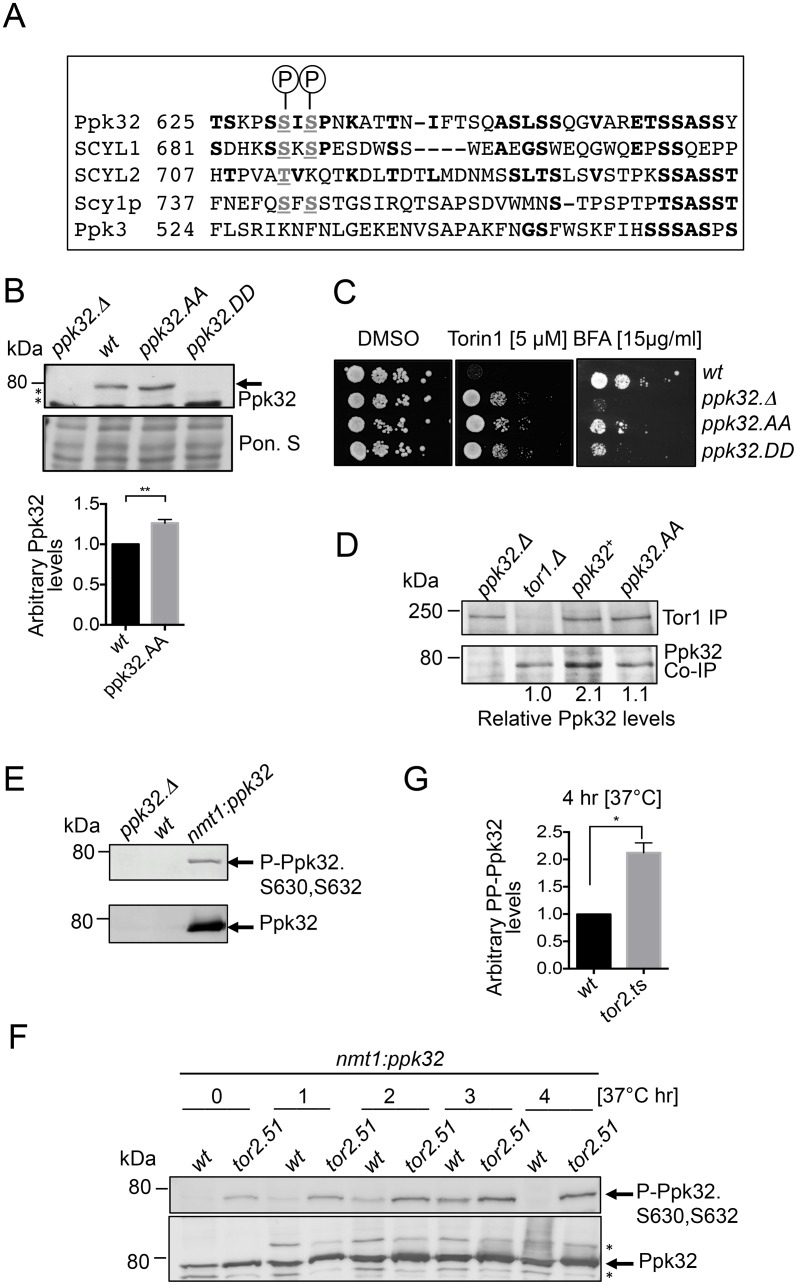
Ppk32 function is regulated by conserved phosphorylation. All strains are leucine autotrophs. (A). Sequence alignment between Ppk32 and other SCYL family kinases. (B) Early exponential cells were grown in rich medium (YES). Samples from non-stressed cultures were taken for Western blot analysis of Ppk32 level. Ponceau S staining was used as a loading control, * indicates a background band. (C) Growth assay. Exponentially YES-grown cells of indicated strains were spotted in 10-fold serial dilution onto the indicated media. (D) Tor1 immuno-precipitations first 3 lanes (Fig 1) are shown again for comparison. Soluble proteins were extracted from exponentially grown strains. Tor1 immuno-precipites and Ppk32 co-immuno-precipites were assessed from the same gels. The relative levels of specific Ppk32 immuno-precipitation are shown. (E-F) Early exponential indicated strains were grown in minimal media (EMMG) to achieve Ppk32 over-expression. Shifted from 28°C to 37°C to induce heat stressed (F) Western blot analysis of phosphorylated S630 and S632 of Ppk32 and total Ppk32 levels. (G) Quantification of relative Ppk32 S630 and S632 phosphoryaliton after 4 hours heat stress.

Global phosphoproteomic approaches have revealed that Ppk32 is phosphorylated on the carboxyl terminal Serine 630 and Serine 632 [49–51]. These phosphorylation sites are conserved throughout the SCYL family of kinases (Fig 6A) but are not present in the other fission yeast SCYL homolog Ppk3 that has no impact upon sensitivity to Torin1 (Fig 1C). To assess the significance of phosphorylation on Ser630 and Ser632 upon the function of *S*. *pombe* Ppk32, the endogenous *ppk32*^+^ locus was manipulated to simultaneously mutate both sites to either alanine to block signalling or to aspartic acid to mimic constitutive phosphorylation. Ppk32 could not be detected in the *ppk32*.*DD (ppk32*.*S630D-S632D)* mutant whereas levels were modestly enhanced in *ppk32*.*AA (ppk32*.*S630A-S632A)* cells (Fig 6B). Both mutations elevated tolerance to Torin1 (Fig 6C). The low Ppk32 levels in the *ppk32*.*DD* mutants are reminiscent of a *ppk32*.*Δ* and thereby account for the Torin1 resistance of this mutant. In contrast, the resistance to Torin1 of *ppk32*.*AA* is unlikely to arise from changes in protein level. Interestingly, comparisons with wild type controls revealed that the phosphoblocking mutation *ppk32*.*AA* reduced the amount of Ppk32 that immuno-precipitated with Tor1 (Fig 6D). Thus, phosphorylation at these residues appears to modulate the affinity of Ppk32 for Tor1. In line with the imposed resistance to Torin1, both phosphorylation site mutants conferred sensitivity to BFA induced stress (Fig 6C), to further support the view that phosphorylation is important for protein function.

Next, phospho-specific antibodies that recognise Ppk32 when phosphorylated on both S630 and S632 were generated by Eurogentec (S7A Fig). Consistent with the inability to detect any Ppk32 in the *ppk32*.*DD* mutant (Fig 6B) we were unable to detect Ppk32 phosphorylation in wild type cells (Fig 6E). To facilitate the detection of Ppk32 phosphorylation *ppk32*^*+*^ was over-expressed from the *nmt1* promoter [52] (Fig 6E) in wild type cells and TORC1 deficient *tor2*.*ts* mutants that had been subjected to heat stress at 37°C (Fig 6F). Comparison of Ppk32 phosphorylation with Ppk32 total proteins levels in these heat stressed cells revealed that phosphorylated Ppk32 was degraded in wild type cells, but not in the TORC1 deficient *tor2*.*ts* mutant (Fig 6F and 6G & S7B Fig). When considered alongside the TORC1 dependent turnover of wild type Ppk32 (Fig 2A), these data suggest that TORC1 activity promotes the degradation of phosphorylated Ppk32.

### The Ppk32 carboxy terminus is required for function

**Fig 7:**
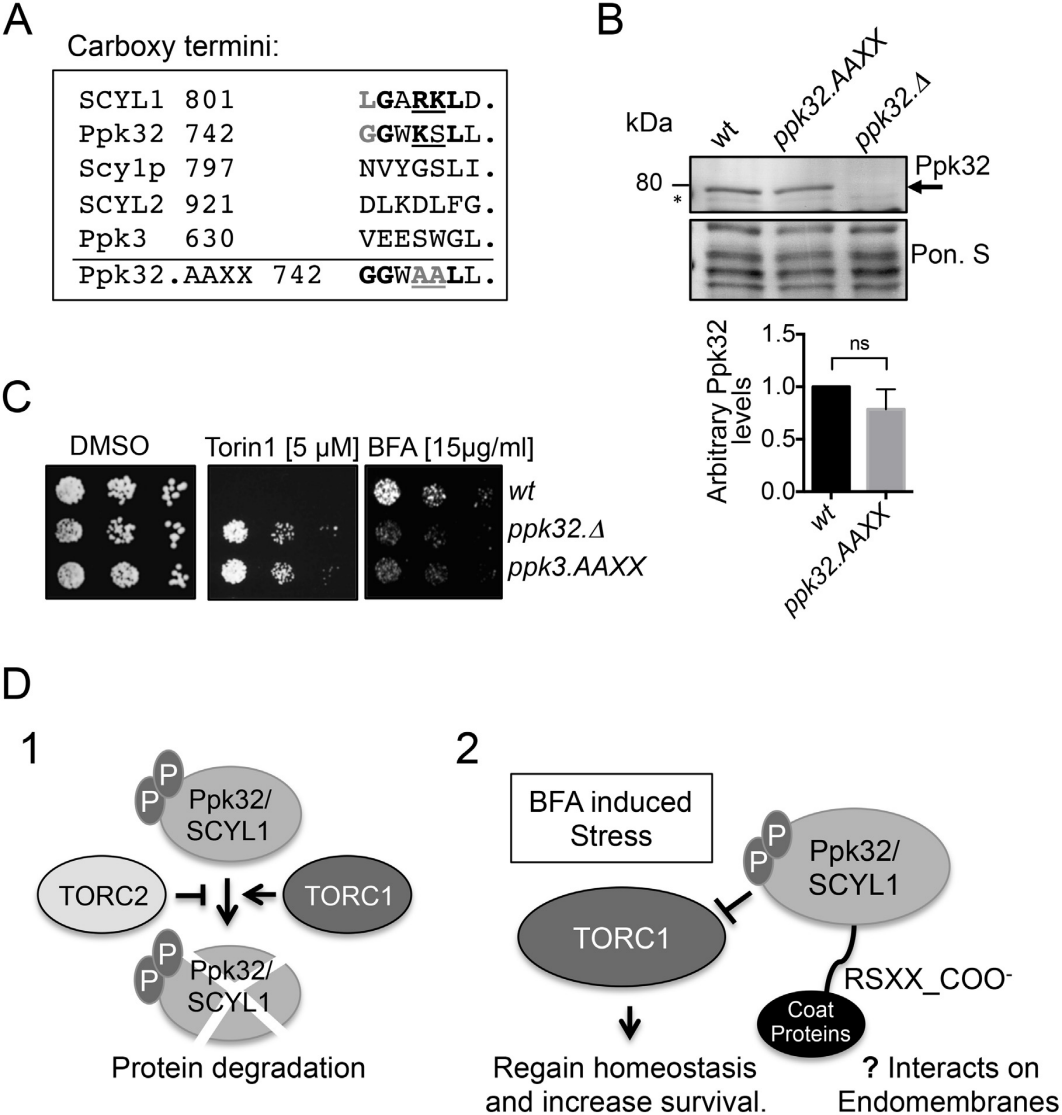
Ppk32 carboxy-terminus is important for protein function. All strains are leucine autotrophs. (A). Sequence alignment of the carboxy terminus of Ppk32 and other SCYL family kinases. (B) Early exponential cells were grown in rich medium (YES). Samples from non-stress cultures were taken for Western blot analysis of Ppk32 level. Ponceau S staining was used as a loading control, * indicates a background band. (C) Growth assay. Exponentially YES-grown cells of indicated strains were spotted in 10-fold serial dilution onto the indicated media. (D) Schematic (1) suggesting that both TORC1 and TORC2 regulate Ppk32 levels. Schematic (2) suggesting that Ppk32 phosphorylation and its carboxy terminus are essential to control TORC1 following BFA induced stress.

The regions adjacent to the Ser630 and Ser632 phosphorylation sites reside in the part of the protein that is most highly conserved in human SCYL1 (Fig 6A). The carboxy terminal RKXX-COO^−^ motif of SCYL1 binds to coatomer (a COP1 protein complex in an ARF independent manner to regulate retrograde transport and Golgi morphology) [22,36]. This SCYL1 RKXX motif and the sourounding sequence is partly conserved in Ppk32 (Fig 7A). Ppk32 has a mono-basic motif followed by a serine in place of the di-basic motif found in SCYL1 (Fig 7A). Non-conventional mono basic motifs have previously been shown to promote binding to COP proteins [53]. To assess the significance of Ppk32 carboxy-terminal sequence Lys745 and Ser746 they were simultaneously mutated to alanine at the native locus to generate the *ppk32*.*L745A-S746A (ppk32*.*AAXX*) allele (Fig 7A). Ppk32 protein levels were unaffected by the presence of the *ppk32*.*AAXX* allele (Fig 7B), however, the mutant gene conferred both resistance to Torin1 and sensitivity to BFA (Fig 7C). Thus, the Ppk32 carboxy-terminal domain of Ppk32 is required for function.

## Discussion

Here we describe a novel control of TORC1 signalling by the SCYL family pseudo-kinase Ppk32 that is essential for survival following BFA induced stress. As the impact of BFA on cell growth that we report here is unlikely to arise from ER Stress (S4A Fig), it probably arises from either Golgi stress or impaired traffic to lysosomes. It is the response to this stress that requires Ppk32 dependent control of TORC1 activity. Interestingly the system appears to involve negative feedback from TOR to Ppk32 as TOR signalling regulates Ppk32 protein levels (summarised in Fig 7D1). At present, it is unclear where in the cell this TOR mediated control of Ppk32 protein levels occurs and where Ppk32 control of TOR signalling takes place.

We show that Ppk32 phosphorylation on two conserved residues is important for protein function. Ppk32 phosphorylation dramatically reduced protein levels and conferred resistance to TOR inhibition. Similarly, despite the increase in protein levels that arises as a consequence of an inability to phosphorylate Ppk32, lack of phosphate on these residues provides resistance to TOR inhibition and highlights the functional significance of these phosphorylation events. Importantly our data suggest that Ppk32 phosphorylation promotes the association of Ppk32 with Tor1.

The impact of Ppk32 on global TOR signalling in non-stressed, steady state conditions is small but measurable, as Ppk32 deficient cells are resistant to Torin1 induced inhibition of TOR signalling (Fig 1C). Our data show how this impact on TOR signalling is dependent on the composition of growth media. A previous report has described an impact of media composition (rather than starvation) on cell growth and chronological lifespan (a process regulated by TOR signalling) to suggest that the steady state level of TOR signalling is regulated by the nutrient composition [54]. We found that Ppk32 deficient cells that were autotrophic for leucine biosynthesis displayed increased resistance to Torin1. Leucine is a well-known activator of TOR signalling in a process that occurs on the endomembranes of vacuoles (yeast lysosomes) [55]. TORC1 concentrates on vacuolar endomembranes [20]. Activation of TORC1 by the addition of leucine may be more efficient in *ppk32*.*Δ* cells because these cells lack an apparent inhibitor of TOR signaling. However, whether phosphorylated Ppk32 interacts with vacuolar TORC1 is unclear at present.

Ppk32 shares sequence similarity with both human SCYL family kinases. However, the requirement of the Ppk32 C-terminal for survival following BFA induced stress and the fact that lack of *ppk32*^*+*^ expression has no impact on endocytosis (a SCYL2 function) suggests that, despite slightly higher level of homology to SCYL2, Ppk32 appears to be a functional SCYL1 homologue in the context of BFA induced stress.

It is unclear whether Ppk32 localizes to the Golgi or COPI budding vesicles. In fission yeast Golgi localisation is seen as small cytoplasmic foci [56]. We did observed small foci in the cytoplasm, however the molecular composition of these is unclear at present. A systematic screen of protein localizations in fission yeast, reported the recruitment of an over-expressed Ppk32 protein to Golgi-like structures [57], suggesting that Ppk32 may localize to the Golgi in fission yeast. Interestingly The RSXX carboxy-terminal motif of Ppk32 is important for cells to tolerate BFA induced stress. Because mTORC1 is localized to the Golgi [58] and to lysosomes [55], phosphorylated Ppk32 may facilitate control of TORC1 signalling on either endomembrane system, however this is unclear at present. Localized control of TORC1 activity would explain why a lack of *ppk32*^*+*^ expression had no impact on TORC1 control of Maf1 (cytoplasmic protein) phosphorylation. Future EM based localisation with specific Ppk32 antibodies will determine whether Ppk32 resides on the Golgi and on vacuoles.

SCYL1 regulates both retrograde trafficking of COPI-coated vesicles from Golgi to ER and transport between Golgi stacks [22,35]. Recently SCYL1 was shown to act as a scaffold that supports the interaction between ARFs and COPI complexes [36]. BFA inhibits the GEFs for class II ARFs [16,17] to release ARF into the cytosol. We show that Ppk32 controlled TORC1 inhibition following BFA induced stress was essential for survival. This Ppk32 controlled reduction in TORC1 signalling will slow down cell metabolism and proliferation. This impairment of metabolism is likely to assist recovery from stress to the membrane traffic system and therefore support survival of BFA stressed cells (summarised in Fig 7D2). We show that a simultaneous reduction of TOR activity, by combining treatment with BFA with additional TOR inhibitors, enhanced cell recovery from BFA induced stress, irrespective of Ppk32 status. Interestingly, one hallmark of cancer is an alteration of the glycosylation landscape that arises from modified Golgi function. Our findings suggest that therapeutic rapamycin treatment to reduce TORC1 signalling and proliferation of cancer cells that are experiencing inherent Golgi stress, might actually have the unintended consequence of enhancing cell survival. It is notable therefore that the control of phosphorylation at the sites in SCYL1, that are equivalent to those identified in fission yeast Ppk32, would be predicted to reduce SCYL1 protein levels and so increase cell death and treatment of cancer types with inherent Golgi stress.

## Methods

### Cell cultures and strains

Strains used in this study are listed in Table 1. Cells were exponentially grown for 36 hr at 28°C in YES [59] 0.15 mg ml^−1^ adenine hemisulphate, uracil, L-leucine and histidine (each), or for 48 hr at 28°C either EMM-G or EMM-P [30]. L-leucine was added to a final concentration of 150 μg ml^−1^. For western blots and growth assays cells were grown to a density of 2×10^6^ cells ml^−1^. For nutrient starvation cells were grown in YES and at the density of 2×10^6^ cells ml^−1^ cultures were filtered into sporulation liquid SPL [60]. For mating assays, cells were grown to a density of 2×10^6^ mixed in a ratio of 1:1 *h*^*+*^:*h*^*−*^ 1.5x10^6^ cells of each strain, washed with water and spotted onto SPA (SPL with 30 g l^−1^ agar). Mating efficiency was calculated as [61]. For halo assays, cells were grown to a density of 2×10^6^ and mixed with *h*^*−*^ cell in ratio: 200:1, washed with water and spotted onto MSA [48].

**Table 1:** Yeast strains used in this study.

*Strain number*	*genotype*	*source*
JP164	*ppk3*::*ura4*^*+*^ *leu1*.*32*	*ppk3*::*ura4*^*+*^ From [34]
JP305	*h*^*−*^ *leu1*.*32*	Lab stock
JP755	*fkh1*::*ura4*^*+*^ *leu1*.*32*	*fkh1*::*ura4*^*+*^ from [29]
JP1293	*h*^*−*^ *tor1*::*ura4*^*+*^ *ura4*.*d18 leu1*.*32*	Lab stock
JP1960	*h*^*−*^ *ppk32*::*ura4*^*+*^ *ura4*.*d18 leu1*.*32*	*ppk32*::*ura4*^*+*^ From [34]
JP2047	*h*^*−*^ *ppk32*::*ura4*^*+*^ *ura4*.*d18*	This study
JP2131	*maf1*.*PK*:*kanMx6 leu1*.*32*	This study
JP2404	*ade6*.*M210/ade6*.*M216 mat2*,*3*::*leu2/mat2*,*3*::*leu2 leu1*.*32/leu1*.*32 ura4*.*d18/ura4*.*d18 mat1-MC*::*ura4*^*+*^*/MAT1 MAT1/mat1-P*	[48]
JP2450	*ppk4*::*ura4*^*+*^ *ura4*.*d18 leu1*.*32*	*ppk4*::*ura4*^*+*^ From [34]
JP2458	*maf1*.*PK*:*kanMx6 ppk32*::*ura4*^*+*^ *ura4*.*d18 leu1*.*32*	This study
JP2480	*tor2*.*51*:*ura4*^*+*^ *ppk32*::*ura4*^*+*^ *ura4*.*d18 leu1*.*32*	This study
JP2495	*ste20*::*kanMx6 leu1*.*32*	*ste20*::*kanMx6* from [64]
JP2502	*ppk4*::*ura4*^*+*^ *ppk32*::*ura4*^*+*^ *ura4*.*d18 leu1*.*32*	This study
JP2503	*h*^*+*^ *ppk32*::*ura4*^*+*^ *ura4*.*d18 leu1*.*32*	This study
JP2504	*h*^*−*^ *leu1*.*32 + pRep1—nmt1*:*leu1*^*+*^	This study
JP2512	*h*^*−*^ *leu1*.*32 + pRep1 –nmt1-ppk32*:*leu*^*+*^	This study
JP2514	*h*^*+*^ *leu1*.*32*	This study
JP2659	*tor1*.*I1816T leu1*.*32*	*tor1*.*I1816T* from [39]
JP2709	*tor2*.*51*:*ura4*^*+*^ *ura4*.*d18 leu1*.*32*	*tor2*.*51*:*ura4*^*+*^ from [5]
JP2711	*tor1*.*I1816T ppk32*::*ura4*^*+*^ *ura4*.*d18 leu1*.*32*	This study
JP2882	*ppk3*::*ura4*^*+*^ *ppk32*::*ura4*^*+*^ *leu1*.*32*	This study
JP3061	*ppk32*.*S630D-S632D leu1*.*32*	This study
JP3116	*ppk32*.*S630A-S632A leu1*.*32*	This study
JP3094	*ppk32*.*AAXX leu1*	This study

The *ppk32* open reading frame was amplified by PCR from genomic *wt* DNA, sequenced and cloned into Nde1 of the pRep1 plasmid to facilitate Ppk32 overexpression from the *nmt1* promoter [52]

### Microscopy

In order to analyse vacuoles morphology, cells were grown in YES to the density of 1.8 × 10^6^ cells ml^−1^ before FM4-64 dye 5mg/ml was added to the culture in ratio: 1:10 000 (dye:culture, v/v) and cells were incubated further. After 30 min, stained vacuoles in live cells were analyzed under the microscope. For each culture at least 500 vacuoles were measured.

### Biochemistry

Total protein extracts were prepared by TCA precipitation [62].

For Tor1 IP, 3x10^8^ cells (culture density 2,4x10^6^ cells ml^−1^) were harvested and re-suspended in IP buffer (50 mM HEPES pH 7.5, 150 mM NaCl, 0,1% CHAPS, 0,05% Tween20, 50 mM L-arginine, 50 mM L-glutamic acid, 50 mM NaF, 2 mM NaVO_4_, 60 mM sodium glycerol phosphate, 5 mM NEM, 1 mM PMSF, 1 mM DTT and Sigma protease inhibitor cocktail) and broken in a FastPrep using glass beads. The cell extract was spun (10 000 rpm, 10 min, 4°C) and supernatant was incubated with Invitrogen protein Dynabeads pre-coupled with anti-Tor1, anti-V5 (PK) or anti-myc antibodies for 20 min at 4°C. Beads were then washed three times with IP buffer and heated to 80°C for 10 min to elute the proteins. Samples were loaded on a SDS-PAGE gel and subsequently processed as total protein extracts.

Proteins were detected using the following antibodies: 1:2000 anti-Ppk32 (polyclonal antibodies raised by Eurogentec Ltd to the following peptide PSEARTPSVQPANRR in rabbits), 1:2000 anti-V5 (anti-PK) (AbD Serotec), 1:1000 anti-phospho Ser546 Gad8 [26], 1:100 Gad8 [26], 1:1000 anti-Tor1 [39], 1:2000 anti-PAS (Cell Signaling Technology), 1:2000 anti-Rps6 (Abcam), anti-phospho-Ser630 S632 Ppk32 (generated by Eurogentec). Alkaline phosphatase coupled secondary antibodies were used for all blots, followed by direct detection with NBT/BCIP (VWR International Ltd.) substrates on PVDF membranes (Millipore). Ponceau S staining was used as a loading control [63]. Relative protein levels were quantified in ImageJ and GraphPad Prism was used to calculated significant differences. Asterisks represent statistical significance (p<0.05) as determined by a Student’s t-test.

## Supporting Information

S1 Fig Ppk32 deficient cells are proficient for Torin1 uptake. All strains are leucine autotrophs. (A,B,D) Western blot analysis: Early exponential cells were grown in rich medium (YES) with addition of increasing Torin1 concentration (D). Samples from indicated cultures were taken for Western blot analysis of Ppk32 level, * indicates a background band. (C) Growth assay. Exponentially YES-grown cells of indicated strains were spotted in 10-fold serial dilution onto the indicated media. (E) Early exponential cells were grown in rich medium (YES) or minimal medium (EMMG). Samples from non-stressed cultures were taken for Western blot analysis of Ppk32 levels. Phos-tag was added to the gel to reveal that Ppk32.PK is phosphorylated. Ponceau S staining was used as a loading control.
(TIF)

S2 Fig Heat stress regulates TORC1. All strains are leucine autotrophs. (A,B) Early exponential cells were grown in rich medium (YES) and shifted into 37°C (A) or treated with Rapamycin (B). Samples were taken for Western blot analysis of Maf1.PK levels of phosphorylation. Ponceau S staining was used as a loading control. (C) Early exponential cells were grown in rich medium (YES). Samples from non-stressed cultures were taken for Western blot analysis of Ppk32 levels. Ponceau S staining was used as a loading control.
(TIF)

S3 Fig Ppk32 inhibits growth and TOR signalling. (A, B) Early exponential cells expressing *ppk32* from the *nmt1* promoter or transformed with an empty vector were grown in minimal medium (EMMG) with (low) or without (high) the addition of 10 μM thiamine. (A) Growth assay. Early exponential cultures were spotted in 10-fold dilution onto the indicated media (low = with thiamine) or (high = without thiamine). (B) Samples were taken for Western blot analysis of Ppk32 level, Maf1.PK and Ser546 Gad8 phosphorylation. Ponceau S staining was used as a loading control.
(TIF)

S4 Fig BFA sensitivity of fission yeast does not arise from ER stress. (A) Growth assay. Exponentially YES-grown cells of indicated strains were spotted in 10-fold serial dilution onto the indicated media to assess cell fitness.
(TIF)

S5 Fig Torin1 promotes cell growth in the presence of BFA. (A,B) Growth assay. Exponentially YES-grown cells of indicated strains were spotted in 10-fold serial dilution onto the indicated media to assess cell fitness.
(TIF)

S6 Fig *ppk32*.*Δ* mutant cells are autophagy proficient. (A) Western blot analyses of Ppk32. Early exponential cells were grown in rich medium (YES). Samples from indicated cultures were subjected to Western blot, * indicates a background band. (B) Growth assay. Exponentially grown cells were deprived of nitrogen by shifting from MSL+Nitrogen into MSL-Nitrogen medium and spotted in 10-fold serial dilution onto YES plates at the indicated time points.
(TIF)

S7 Fig Phospho-Ppk32.S630-S632 antibodies are phosphor-specific. (A, B) Western blot analyses of phospho-Ppk32. Early exponential cells expressing *ppk32* from the *nmt1* promoter were grown in minimal medium (EMMG) without the addition of thiamine for 20 hr (high expression). (A) Soluble total protein extracts were exposed to Lambda phosphatase with or without the addition of phosphatase inhibitors. (B) A *tor2*.*51* mutant were shifted to 37°C for 3 hr to inactivate TORC1. The level of phosphorylated Ppk32 increases when TORC1 is inactivated.
(TIF)
