# Supplementary figures and images for: Fission Yeast SCYL1/2 Homologue Ppk32: A Novel Regulator of TOR Signalling That Governs Survival during Brefeldin A Induced Stress to Protein Trafficking
Source: PLoS Genet. 2016 May 18;12(5):e1006041. doi: 10.1371/journal.pgen.1006041 (PMC4871519; doi:10.1371/journal.pgen.1006041)

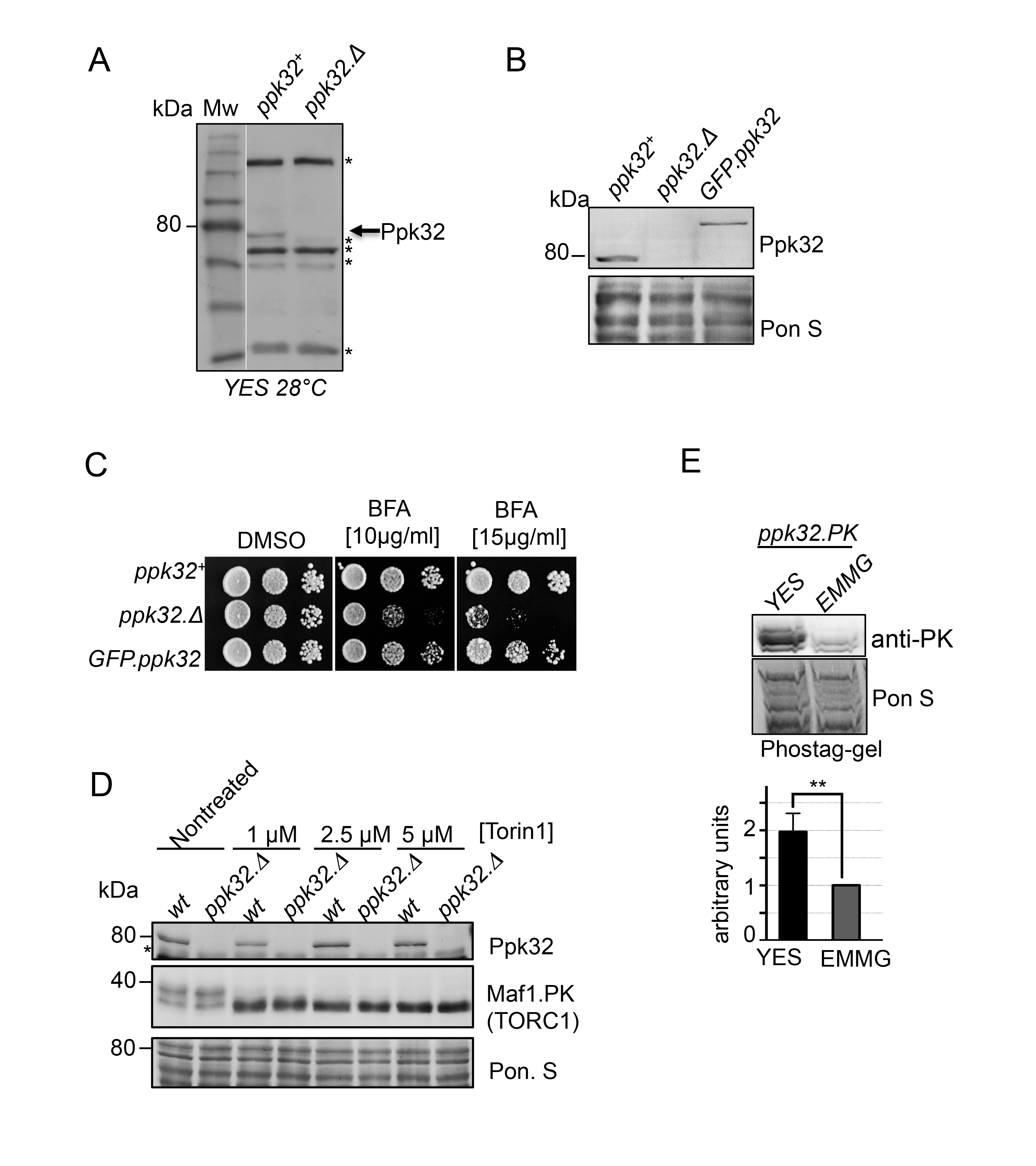

Supplement: S1 Fig — All strains are leucine autotrophs. (A,B,D) Western blot analysis: Early exponential cells were grown in rich medium (YES) with addition of increasing Torin1 concentration (D). Samples from indicated cultures were taken for Western blot analysis of Ppk32 level, * indicates a background band. (C) Growth assay. Exponentially YES-grown cells of indicated strains were spotted in 10-fold serial dilution onto the indicated media. (E) Early exponential cells were grown in rich medium (YES) or minimal medium (EMMG). Samples from non-stressed cultures were taken for Western blot analysis of Ppk32 levels. Phos-tag was added to the gel to reveal that Ppk32.PK is phosphorylated. Ponceau S staining was used as a loading control. (TIF) [file pgen.1006041.s001.tif]

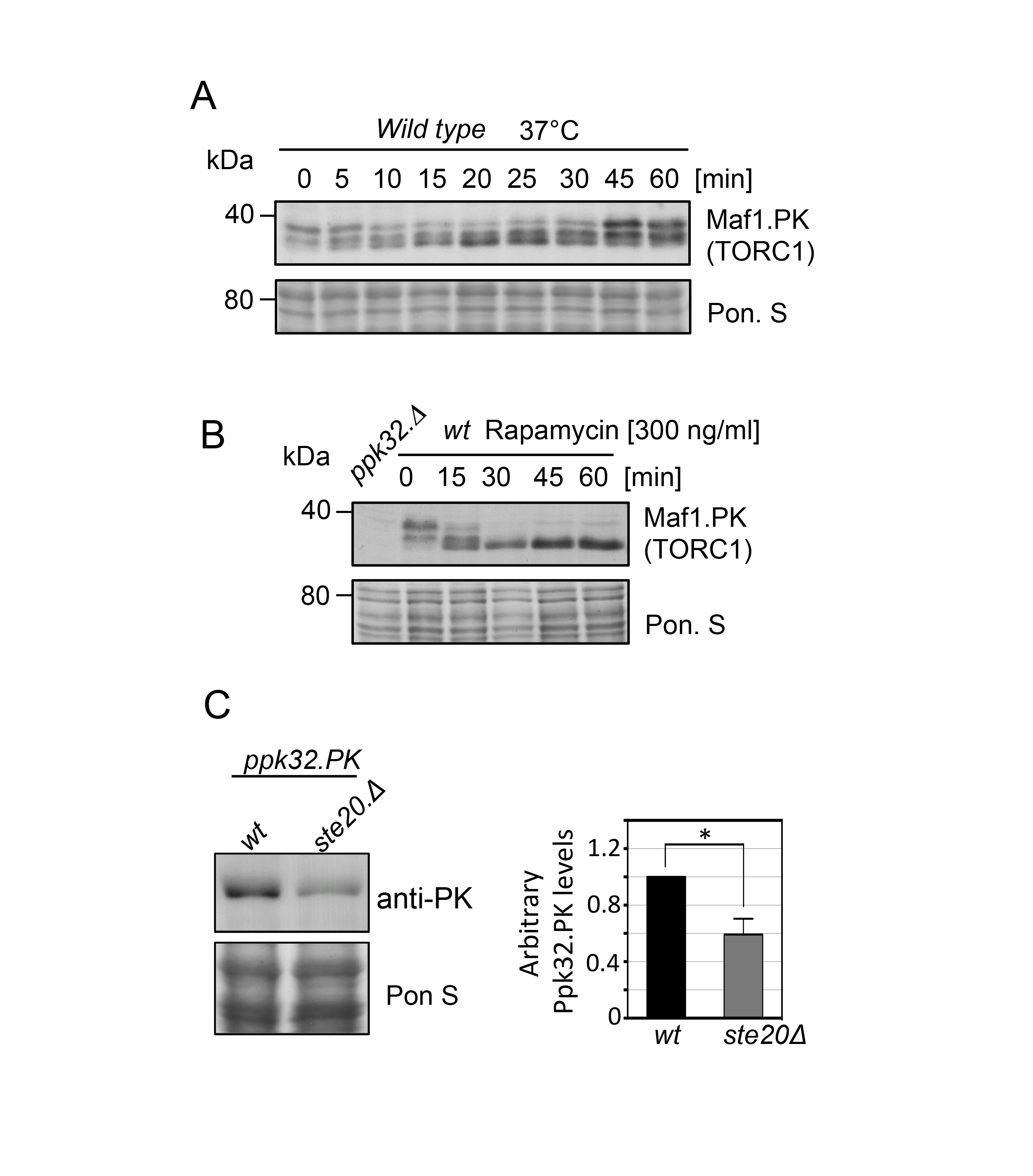

Supplement: S2 Fig — All strains are leucine autotrophs. (A,B) Early exponential cells were grown in rich medium (YES) and shifted into 37°C (A) or treated with Rapamycin (B). Samples were taken for Western blot analysis of Maf1.PK levels of phosphorylation. Ponceau S staining was used as a loading control. (C) Early exponential cells were grown in rich medium (YES). Samples from non-stressed cultures were taken for Western blot analysis of Ppk32 levels. Ponceau S staining was used as a loading control. (TIF) [file pgen.1006041.s002.tif]

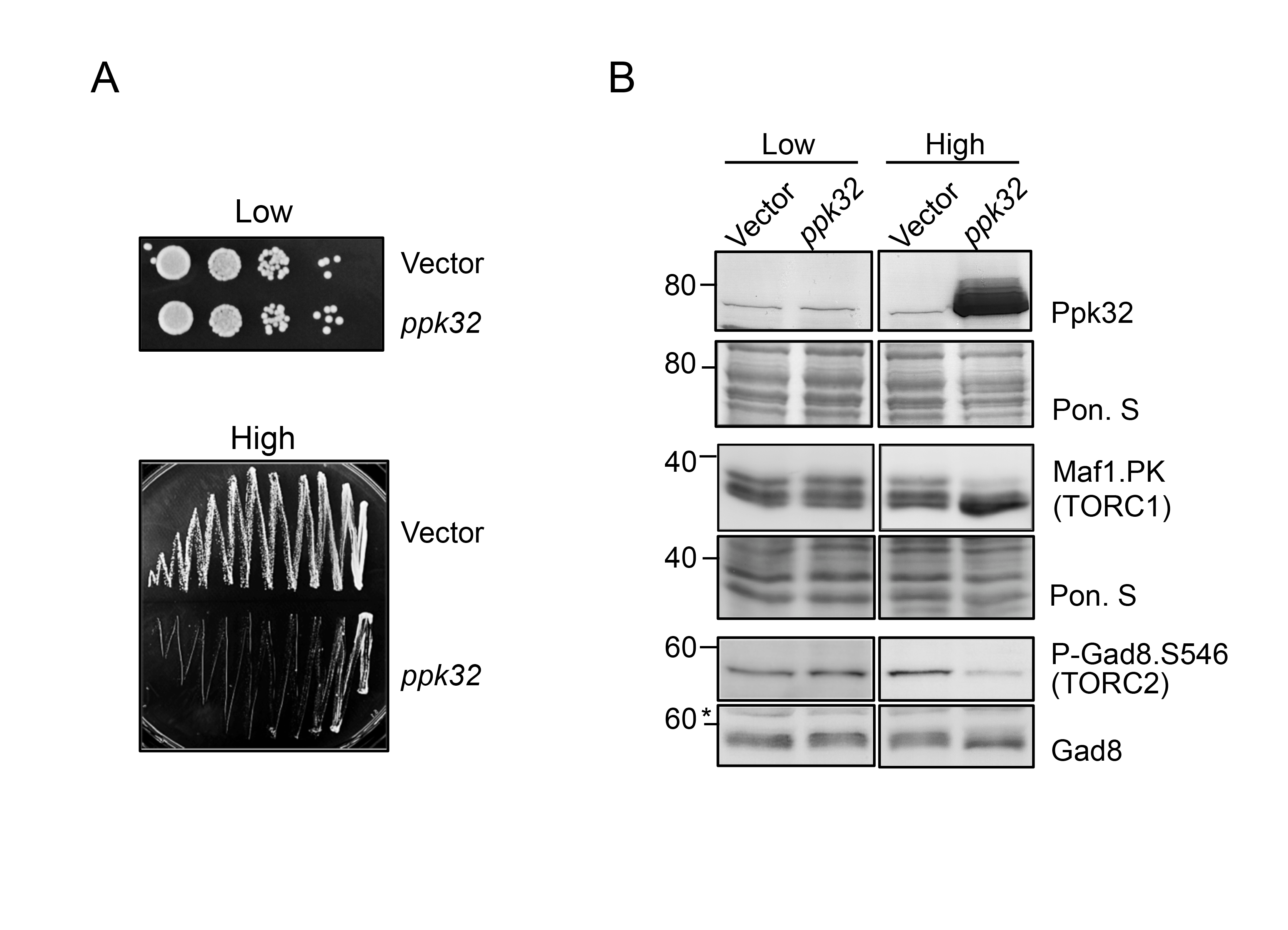

Supplement: S3 Fig — (A, B) Early exponential cells expressing ppk32 from the nmt1 promoter or transformed with an empty vector were grown in minimal medium (EMMG) with (low) or without (high) the addition of 10 μM thiamine. (A) Growth assay. Early exponential cultures were spotted in 10-fold dilution onto the indicated media (low = with thiamine) or (high = without thiamine). (B) Samples were taken for Western blot analysis of Ppk32 level, Maf1.PK and Ser546 Gad8 phosphorylation. Ponceau S staining was used as a loading control. (TIF) [file pgen.1006041.s003.tif]

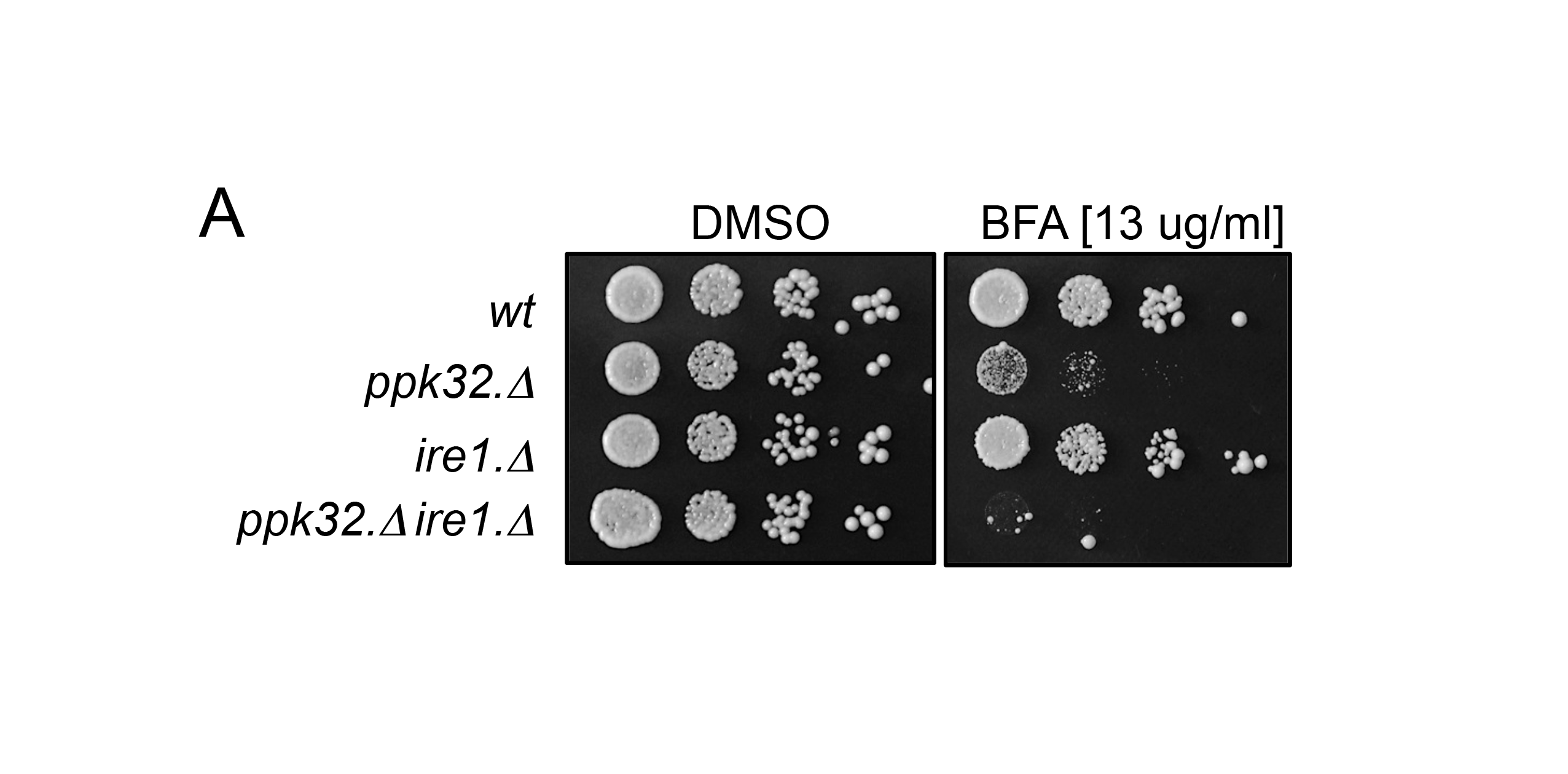

Supplement: S4 Fig — (A) Growth assay. Exponentially YES-grown cells of indicated strains were spotted in 10-fold serial dilution onto the indicated media to assess cell fitness. (TIF) [file pgen.1006041.s004.tif]

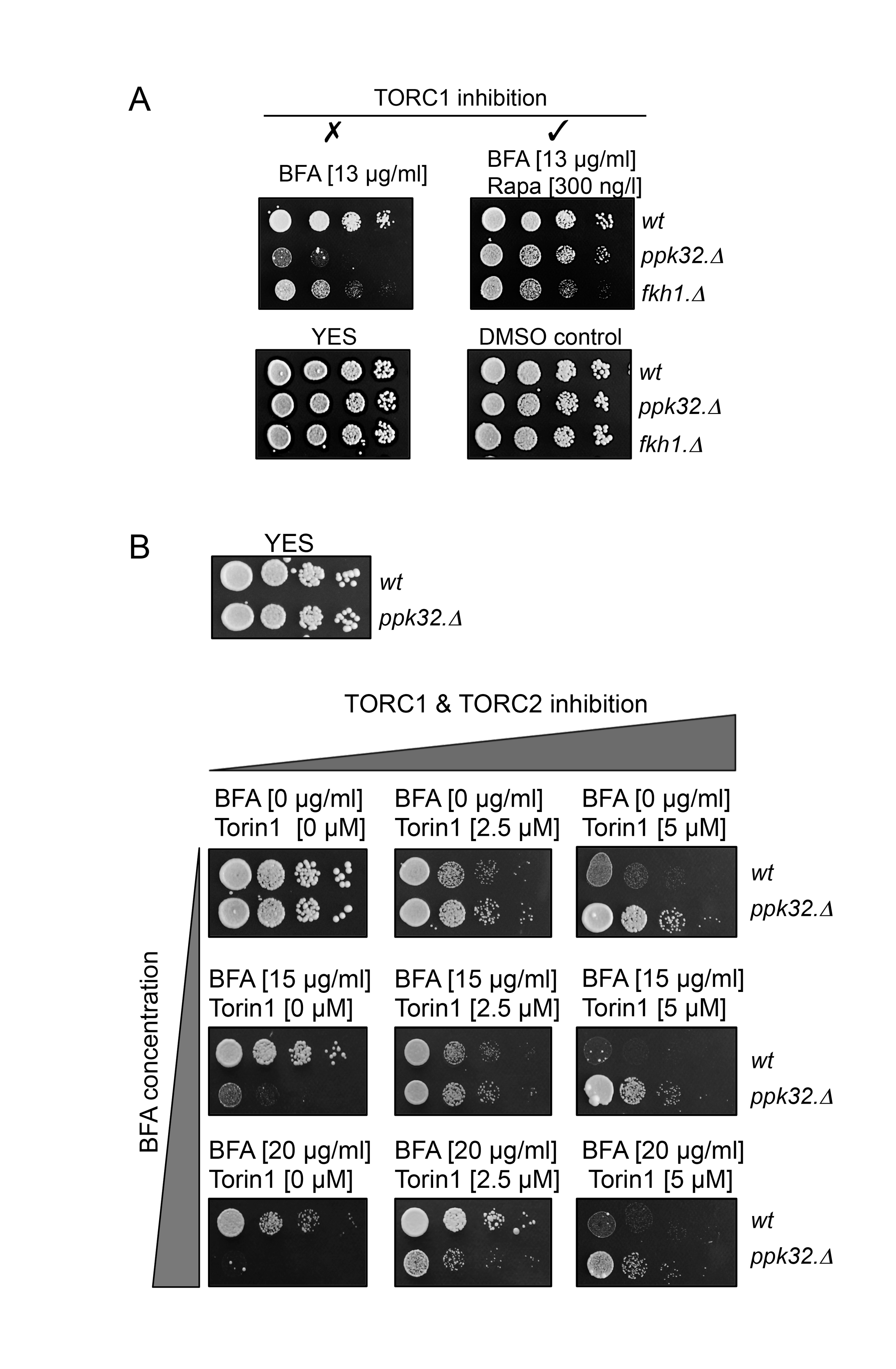

Supplement: S5 Fig — (A,B) Growth assay. Exponentially YES-grown cells of indicated strains were spotted in 10-fold serial dilution onto the indicated media to assess cell fitness. (TIF) [file pgen.1006041.s005.tif]

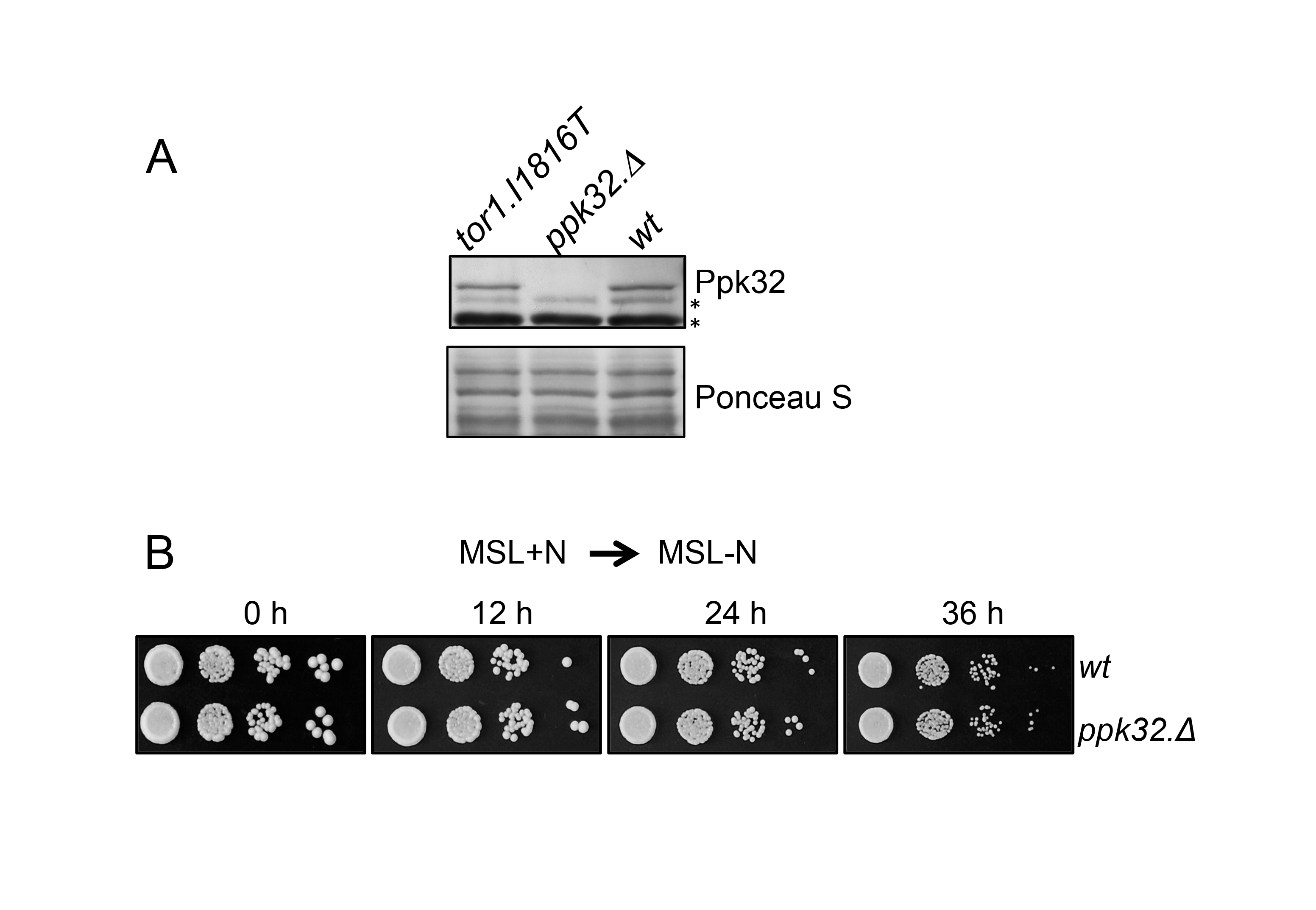

Supplement: S6 Fig — (A) Western blot analyses of Ppk32. Early exponential cells were grown in rich medium (YES). Samples from indicated cultures were subjected to Western blot, * indicates a background band. (B) Growth assay. Exponentially grown cells were deprived of nitrogen by shifting from MSL+Nitrogen into MSL-Nitrogen medium and spotted in 10-fold serial dilution onto YES plates at the indicated time points. (TIF) [file pgen.1006041.s006.tif]

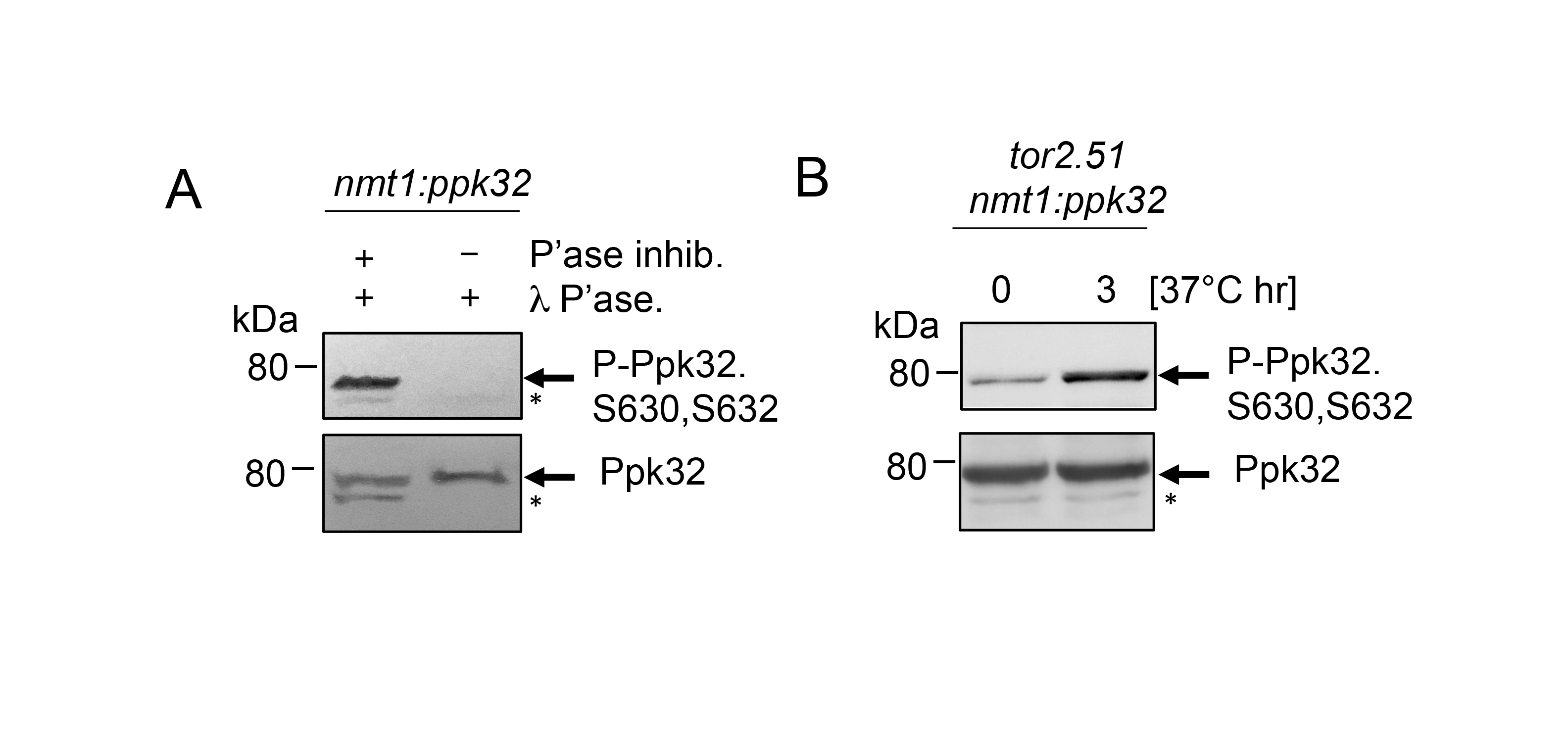

Supplement: S7 Fig — (A, B) Western blot analyses of phospho-Ppk32. Early exponential cells expressing ppk32 from the nmt1 promoter were grown in minimal medium (EMMG) without the addition of thiamine for 20 hr (high expression). (A) Soluble total protein extracts were exposed to Lambda phosphatase with or without the addition of phosphatase inhibitors. (B) A tor2.51 mutant were shifted to 37°C for 3 hr to inactivate TORC1. The level of phosphorylated Ppk32 increases when TORC1 is inactivated. (TIF) [file pgen.1006041.s007.tif]
